# Effect of Chronic Blood Transfusion on Biomarkers of Coagulation Activation and Thrombin Generation in Sickle Cell Patients at Risk for Stroke

**DOI:** 10.1371/journal.pone.0134193

**Published:** 2015-08-25

**Authors:** Hyacinth I. Hyacinth, Robert J. Adams, Charles S. Greenberg, Jenifer H. Voeks, Allyson Hill, Jacqueline M. Hibbert, Beatrice E. Gee

**Affiliations:** 1 Department of Pediatrics Hematology/Oncology, Emory University School of Medicine, Atlanta, GA, United States of America; 2 Stroke Centre, Department of Neurology, Medical University of South Carolina, Charleston, SC, United States of America; 3 Department of Hematology, Medical University of South Carolina, Charleston, SC, United States of America; 4 Department of Biology, College of Charleston, Charleston, SC, United States of America; 5 Department of Microbiology, Biochemistry and Immunology, Morehouse School of Medicine, Atlanta, GA, United States of America; 6 Department of Pediatrics and Cardiovascular Research Institute, Morehouse School of Medicine, Atlanta, GA, United States of America; 7 Children’s Healthcare of Atlanta, Atlanta, GA, United States of America; Sickle Cell Unit, JAMAICA

## Abstract

Hypercoagulability in sickle cell disease (SCD) is associated with multiple SCD phenotypes, association with stroke risk has not been well described. We hypothesized that serum levels of biomarkers of coagulation activation correlate with high transcranial Doppler ultrasound velocity and decreases with blood transfusion therapy in SCD patients. Stored serum samples from subjects in the Stroke Prevention in Sickle Cell Anemia (STOP) trial were analyzed using ELISA and protein multiplexing techniques. 40 subjects from each treatment arm (Standard Care [SC] and Transfusion [Tx]) at three time points—baseline, study exit and one year post-trial and 10 each of age matched children with SCD but normal TCD (SNTCD) and with normal hemoglobin (HbAA) were analyzed. At baseline, median vWF, TAT and D-dimer levels were significantly higher among STOP subjects than either HbAA or SNTCD. At study exit, median hemoglobin level was significantly higher while median TCD velocity was significantly lower in Tx compared to SC subjects. Median vWF (409.6 vs. 542.9 μg/ml), TAT (24.8 vs. 40.0 ng/ml) and D-dimer (9.2 vs. 19.1 μg/ml) levels were also significantly lower in the Tx compared to the SC group at study exit. Blood levels of biomarkers coagulation activation/thrombin generation correlated positively with TCD velocity and negatively with number of blood transfusions. Biomarkers of coagulation activation/thrombin generation were significantly elevated in children with SCD, at high risk for stroke. Reduction in levels of these biomarkers correlated with reduction in stroke risk (lower TCD velocity), indicating a possible role for hypercoagulation in SCD associated stroke.

## Introduction

Sickle cell disease (SCD) is a significant public health problem, affecting over 100,000 people in the United States (US). It is a huge burden to the healthcare system and a significant source of health disparity [1–4]. The most common form, Hemoglobin SS or sickle cell anemia (SCA) is due to a point mutation in both human β-globin genes leading to the substitution of valine for glutamic acid at the 6^th^ positions of both β-globin chains [5]. Hypercoagulability has been described in SCD [6, 7], and been associated with complications of SCD, such as acute chest syndrome (ACS) and vaso-occlusive episodes [8, 9]. Global activation and increased serum levels of pro-thrombotic factors and/or mediators have been described, which may be triggered by erythrocyte microparticles and inflammatory mediators [9, 10]. We have previously shown that children with SCD, at high risk for stroke, demonstrated by a high transcranial Doppler ultrasound (TCD) velocity, have significantly elevated serum levels of platelet derived growth factor AA [11] and total plasminogen activator inhibitor 1 [12]. Elevated baseline levels of both biomarkers were associated with high risk of incident stroke or decreased probability of stroke free survival, respectively [11, 12]. A high TCD velocity, ≥200cm/s [13], the severity of cerebral vasculopathy [14–16], and presence of extracranial vasculopathy [17] are among the recognized risk factors for development of stroke in children with SCD. The role of coagulation activation in SCD associated stroke is still not well-understood.

Chronic packed red blood cell (PRBC) transfusion is currently the main modality for primary [18] and secondary [19] stroke prevention in children with SCD. Although a beneficial regimen, chronic PRBC transfusion is associated with potential complications (iron overload and alloimunization); and there is no safe time to discontinue it [20]. Further, when severe cerebral vasculopathy is established, chronic PRBC transfusion may not prevent occurrence of silent cerebral infarcts [21]. It is has been documented that inflammation [22, 23], vascular remodeling [24] and anemia leading to a hyperdynamic cerebral circulation [25, 26] are associated with an increased risk for developing cerebral vasculopathy and stroke in children with SCA. Anemia and inflammation are mitigated by chronic PRBC transfusion, resulting in decreased stroke risk and incidence [18, 22].

The conditions that could potentially initiate and/or propagate the development of cerebral arteriopathy in SCA remain elusive [27]. In addition, the mechanisms by which PRBC transfusions prevent stroke are not well-defined. This study seeks to delineate the possible role of coagulation activation in the pathobiology of stroke in SCD and the effects of red cell transfusion regimen on the coagulation pathway, especially thrombin generation. We hypothesize that serum levels of biomarkers of coagulation activation and thrombin generation are elevated in children with SCD and high TCD velocity. These levels are expected to be significantly reduced in those who received chronic PRBC transfusion compared to those who did not. Further, the lower levels of coagulation activation will be related to the number of transfusions received and hemoglobin levels.

## Sample, Materials and Methods

### Sample and sample processing

This study is an ancillary to the Stroke Prevention Trial in Sickle Cell Anemia (STOP) [18] [NCT00000592] and is an addition to prior studies [11, 12] from our laboratory. It is a longitudinal cross-sectional analysis of stored serum samples from the STOP study.

In brief, the STOP study was a randomized phase III clinical trial of the efficacy of chronic PRBC transfusion for the prevention of stroke in children with SCD and high TCD velocity. A total of 130 children, ages 2–16 years, with SCA and high TCD velocity (≥ 200cm/s) were randomly assigned to received either standard care (observation, n = 67) or chronic PRBC transfusion (n = 63). The study was terminated early because of the overwhelming evidence of benefit to the transfusion arm [18]. Chronic PRBC transfusion resulted in a 92% reduction in stroke incidence. An additional year of post-trial follow up period was carried out. During this period, subjects randomized to the standard care arm were allowed to cross-over and receive PRBC transfusion. Majority of the subjects in the standard care arm crossed over to receive transfusion, while some patients on the transfusion arm stopped receiving transfusion all together; there were subjects in between, who either receive less or more transfusions during this period. All the patients included in this study from the standard care arm crossed over to receive transfusion while none of those from the transfusion arm in this study, completely stopped receiving transfusion at the end of 1 year post-trial.

In this study, the experimental design included only subjects from the STOP study for whom we had serum samples at all 3 time points to ensure that comparisons between time points included the same subjects. Serum samples were obtained from the bio-repository of the STOP study currently maintained at the Medical University of South Carolina (MUSC). Two hundred and forty frozen serum samples were available from 40 subjects in each of the standard care [SC] and transfusion [Tx] arms at baseline or randomization, study exit and 1 year post-trial time points were analyzed using Enzyme Linked Immuno-Sorbent Assay (ELISA) and multiplex analysis. The sample size for this ancillary was smaller than that of the parent study, but sample size estimates based on either a t or F distribution indicated that the sample size of 40 was robust to type 1 error and adequate to achieve a power of 80%.

An additional 10 samples each from age matched children with **S**ickle cell disease and **N**ormal **TCD** velocity (SNTCD) and healthy African American children with normal hemoglobin (HbAA) were assayed for comparison. The samples for the children with SCD and normal TCD, and healthy control were obtained at “steady state” (when subjects were in a normal state of health (not during acute illnesses), four months after the last transfusion (for SCD subjects), from SCD subject not taking hydroxyurea. These samples were obtained in 2008–2009 as part of a study to investigate vascular function in children with SCD.

The original study did not plan for studies that might require platelet poor serum and as such, samples were not rendered platelet poor before storage in the biorepository. Venous blood was drawn and allowed to clot at room temperature, and then shipped to the Medical College of Georgia (MCG)-Hemoglobinopathy laboratory on wet-ice within 48 hours where samples were centrifuged, serum was removed and frozen (−70°C), subsequent sample storage was done at-80°C. Sample processing for Healthy controls, sickle cell patients with normal TCD velocity and that of STOP study participants were similar. Furthermore, the dates on the sample storage tubes for STOP study subjects indicates that samples were for the most part obtained at or in close proximity to the time of TCD velocity measurement.

We assayed for serum levels of thrombin-antithrombin (TAT) complex based on manufacturer’s protocols with conscious awareness of sample types and sample preparation requirements (http://www.abcam.com/thrombin-antithrombin-complex-tat-human-elisa-kit-ab108907.html and http://www.abcam.com/Thrombin-Antithrombin-Complex-TAT-Human-ELISA-Kit-ab108907.pdf). In addition, we also assayed serum levels of von Willibrand factor (vWF), ADAMTS 13 (to enable us define vWF to ADAMTS 13 ratio for as index of functionality with regards to proteolytic cleavage of vWF by ADAMTS 13), D-dimer, P-selectin and fibrinogen.

### Ethics statement

The original STOP study and consent forms and documents were approved by the institutional review boards (IRBs) of the Medical College of Georgia and New England Research Institute. A written signed informed consent was obtained from participants and their caregivers. Approval to conduct this study was obtained from the IRBs of the Medical University of South Carolina (MUSC), Morehouse School of Medicine (MSM) and Children’s Healthcare of Atlanta (CHOA). Informed consent had been obtained from participants and their caregivers during the original clinical trial to allow for subsequent use of the samples by other investigators without the need for re-consenting.

### ELISA assay

Serum levels of vWF and TAT complex were assayed using ELISA at the Morehouse School of Medicine Proteomics core laboratory. Frozen serum samples were allowed to thaw slowly at room temperature, and along with the assay kits (Abcam PLC., Cambridge, MA), warm up to room temperature. All assays were carried out according to the manufacturer’s protocol and were done in duplicate. Absorbance was read using a Softmax Pro (Molecular Devices LLC, Sunnyvale, CA) with concentration of analytes calculated based on a linear curve generated using manufacturer provided standards, using an R^2^ of 0.95 or greater. The concentration of vWF was read in mU/ml and then converted to ng/ml by multiplying with 9.8 to convert to μg/ml and then by 1000 to convert from μg/ml to ng/ml. This conversion was necessary to enable the calculation of vWF to ADAMTS 13 ratio.

### Multiplex assay

Thawed serum samples (same as was used for ELISA assay) were allowed to warm up to room temperature before analysis. This assay measured serum levels of ADAMTS 13, D-dimer, P-selectin and fibrinogen. Samples for estimating ADAMTS 13, D-dimer and P-selectin were diluted 1:100, while they were further diluted to 1:40,000 to allow for the measurement of fibrinogen level. Assays were carried out in duplicates using a customized human cardiovascular disease panel 2 (ADAMTS 13, D-dimer, P-selectin) and 3 (fibrinogen) of antibody immobilized bead kit (Millipore Inc., Billerica, MA). The mean fluorescent intensity (MFI) was read on a BioRad Bioplex reader powered by Luminex (Bio-Rad Inc., Hercules, CA) and concentration calculated using an interpolated 5PL logistic curve generated using manufacturer supplied standard prepared via a serial 1:4 dilution. In addition to ELISA and multiplex data, values for TCD velocity, anthropometric and hematological data were exported to Microsoft Excel and analyzed using IBM SPSS 20 for Windows and GraphPad Prism.

### Data analysis and presentation

Median anthropometric and hematological characteristics of subjects were calculated at each time point. This is presented in Table 1 along with their respective 10^th^ and 90^th^ percentile in brackets. Except for percentage fetal hemoglobin, where a parametric t-test was used (data was normally distributed), a non-parametric t-test was used in all other cases to compare median differences between SC and Tx groups at each time point. Serum levels of the biomarkers were compared at baseline and at study exit between HbAA, SNTCD, SC and Tx using non-parametric analysis of variance (ANOVA) with Dunn’s correction for multiple comparisons. The median serum levels of biomarkers were expressed using box and whisker plots with 10^th^ and 90^th^ percentiles, at baseline and study exit respectively. The total number of transfusions received during the 30 month study period (some of the SC subjects received transfusions for other indications) was determined. This number was then correlated with TCD velocity and serum levels of biomarkers of coagulation activation and thrombin generation at the study exit time point. A two-tailed partial correlation with adjustment for baseline variation in serum biomarker levels was used. Results were expressed as scatter plots with regression lines and their respective correlation coefficients. Paired sample t-test was used for comparison of serum concentration of biomarkers and TCD velocity at baseline and study exit within each study arm (i.e. baseline vs. study exit for SC arm, and so on) and also expressed in a box and whisker plot (S1 and S2 Files.). Finally the median percentage change in serum levels of the biomarkers from baseline was calculated and then compared between SC and Tx. The results of this is presented in a box and whisker plot for the biomarkers with statistically significant difference.

**Table 1:** Showing baseline and study laboratory and anthropometric characteristics of subjects. Values are medians with 10^th^ and 90^th^ percentile in bracket, except for fetal hemoglobin percent which is expressed in means ± SD. Except for percent fetal hemoglobin (standard t-test), all comparisons were done using a Mann-Whitney test (non-parametric t-test). At baseline, there was no significant difference between subjects on all characteristic except hemoglobin level which was significantly higher among those randomized to receive standard care (p = 0.021) those who were to receive transfusion. At study exit, the group that got transfused had significantly higher hemoglobin level (p = 0.006) and lower TCD velocity (p = 0.002) than the group who did not receive transfusion. Note that those randomized to standard care still got some blood transfusion in the course of the study, but analysis was based on intent to treat. At the one year post-trial time point, TCD velocity (p = 0.034) and for an unknown reason, BMI (p = 0.031) were significantly lower among those who were originally randomized to receive transfusion in the STOP study compared to those randomized to SC. Tx = transfusion.

Variable	Baseline		Study Exit		Post-Trial	
	Standard Care	Transfusion	Standard Care	Transfusion	Standard Care	Transfusion
**Sickle Hemoglobin (%)**	88.5 (80.3, 93.9)	89.4 (76.3, 93.5)	35.6 (15.3, 91.6)	30.6 (16.6, 89.6)	35.7 (15.3, 91.5)	30.6 (16.6, 89.9)
**Fetal Hemoglobin (%)**	9.4 ± 5.1	9.1 ± 5.4	3.7 ± 4.6	2.4 ± 4.0	5.2 ± 11.6	2.6 ± 4.0
**Hemoglobin (g/dL)**	7.6 (6.7, 8.9)	7.2 (6.1, 8.5)	8.0 (6.9, 9.3)	8.8 (7.2, 10.4)	8.4 (7.1, 9.9)	8.4 (6.8, 9.7)
**Age (years)**	8.0 (3.1, 12.9)	8.0 (4.0, 13.9)	-	-	-	-
**Height (m)**	1.3 (0.99, 1.5)	1.2 (1.0, 1.6)	-	-	1.5 (1.2, 1.7)	1.4 (1.3, 1.7)
**Weight (kg)**	25.2 (15.0, 39.7)	22.0 (15.6, 43.0)	-	-	38.9 (21.6, 58.3)	33 (24.0, 60.3)
**Body mass index (kg/m** ^**2**^ **)**	15.2 (13.9, 18.7)	15.2 (13.7, 17.6)	-	-	18.4 (14.0, 26.1)	16.1 (14.2, 21.2)
**White blood cell count (x10** ^**3**^ **/ml)**	12 (7.3, 18.0)	10.8 (6.1. 16.3)	12.3 (7.4, 18.6)	11.7 (5.4, 17.7)	10.7 (6.9, 14.7)	11.0 (7.3, 15.9)
**Platelet count (x10** ^**3**^ **/ml)**	399 (280, 549)	359 (253, 503)	356 (241, 471)	334 (184, 454)	381 (216, 595)	377 (257, 487)
**Reticulocyte (%)**	12.3 (7.5, 19.6)	11.6 (6.4, 18.0)	12.7 (7.5, 18.1)	10.9 (3.7, 19.4)	9.6 (6.4, 14.4)	9.5 (5.7, 15.2)
**TCD velocity (cm/s)**	216 (201, 256.6)	214 (202, 255)	203 (144, 244)	157 (133, 236)	189 (120, 266)	166 (121, 220)
**Total No. of Tx received**	-	-	2 (0, 12.8)	34 (17.5, 54.2)	15 (0, 19)	14.5 (0, 20.9)

## Results

### Clinical characteristics of subjects

**Fig 1:**
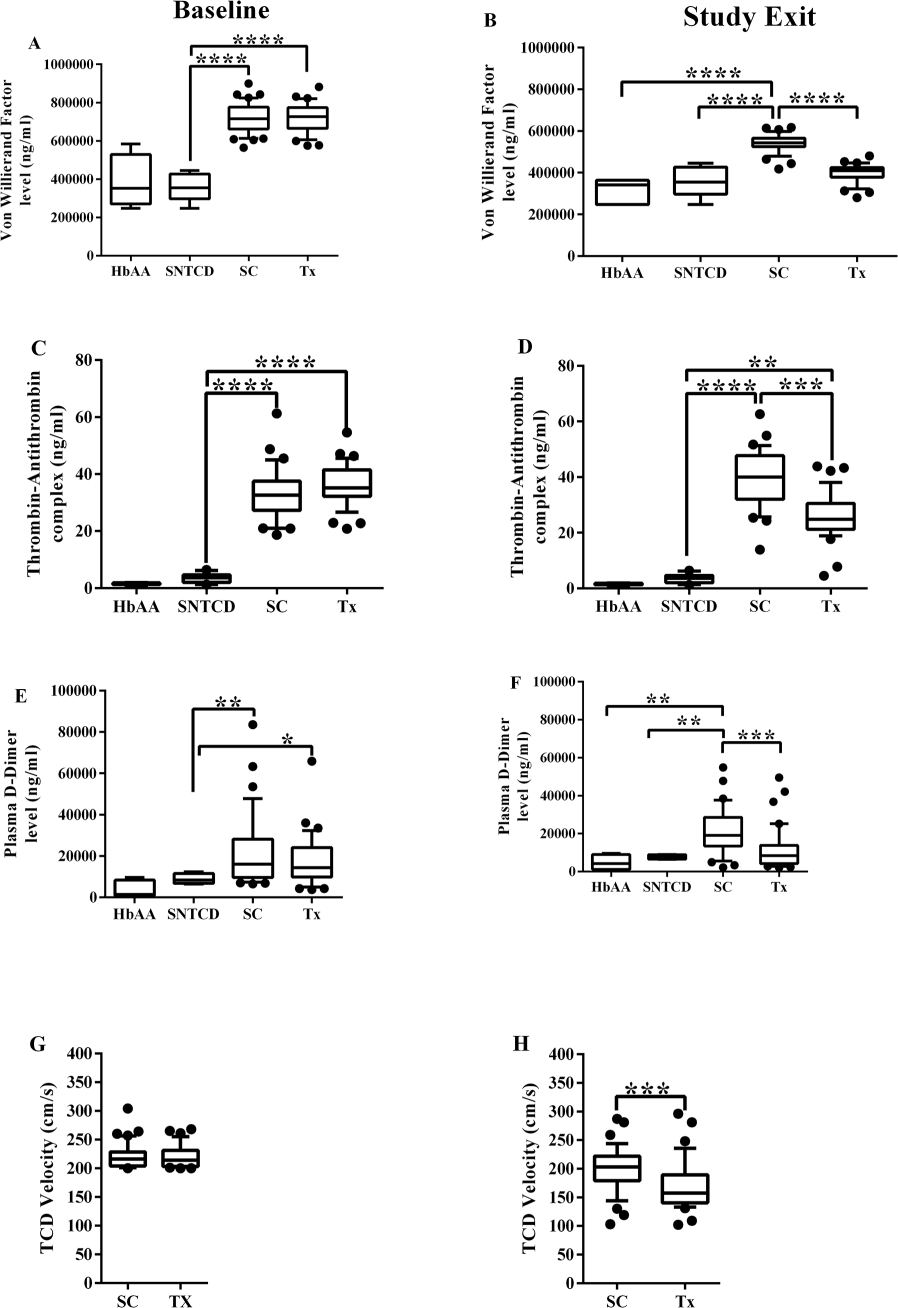
Baseline and study exit values of von Willibrand Factor (vWF), thrombin antithrombin (TAT) complex, D-dimer and transcranial Doppler (TCD) velocity. HbAA, SNTCD, SC and Tx refers to healthy African American children with normal hemoglobin, sickle cell subjects with normal TCD velocity, STOP subjects randomized to the Standard Care arm and sickle cell subjects randomized to the Transfusion arm respectively. * = p <0.05, ** = p <0.01, *** = p <0.001 and **** = p <0.0001.

The clinical characteristics of STOP study subjects whose samples were analyzed are shown in Table 1. Except for median hemoglobin level (7.6 [6.7, 8.9] vs. 7.2 [6.1, 8.5]) mg/dL, which was significantly higher among the Standard Care (SC) (p = 0.021) compared to the Transfusion (Tx) arm, there were no significant differences in baseline characteristics and TCD velocity between study arms. At study exit, median hemoglobin level was significantly higher (8.8 [7.2, 10.4] vs. 8.0 [6.9, 9.3]) mg/dL and median TCD velocity significantly lower (157 [133, 236] vs. 203 [144, 244]) cm/sec among the Tx arm compared to the SC arm, p = 0.006 and 0.002 respectively (Table 1 and Fig 1H). At the one year post-trial time point, those randomized to the Tx arm had significantly lower median TCD velocity (166 [121, 220] vs. 189 [120, 266]) cm/s and body mass index (16.1 [14.2, 21.2] vs. 18.4 [14.0, 26.1]) kg/m^2^ compared to those randomized to the SC arm, p = 0.034 and 0.031 respectively (Table 1).

### Biomarkers of coagulation activation

Despite differences in baseline hemoglobin levels, the subjects in the two treatment arms were well-matched on baseline levels of coagulation biomarkers, with no statistically significant differences in the serum levels of all biomarkers of coagulation activation or thrombin generation. STOP subjects in both SC and Tx groups had significantly higher median levels of vWF, TAT, and D-Dimer than those with SCD and Normal TCD (SNTCD) or healthy controls (HbAA) (Fig 1A, 1C and 1E). While there was no difference in ADAMTS 13 levels between groups (Figure E in S1 File), VWF/ADAMTS 13 ratio was significantly higher at baseline for randomized subjects than for either SNTCD or HbAA groups (Figure G in S1 File). Additionally, the median level of sP-selectin among SC arm was significantly higher than among HbAA (Figure C in S1 File).

At study exit, subjects randomized to the Tx arm had significantly lower median vWF levels than those in the SC arm (409.6 [322.3, 446.2] vs.542.9 [479.6, 597.9]) μg/ml, p <0.0001. But there was no statistically significant difference in median vWF levels between Tx arm and either SNTCD or HbAA subjects at study exit (Fig 1B). As was the case at baseline, the serum vWF level at exit was significantly higher among the SC arm than either SNTCD or HbAA, p <0.0001. Similarly, median serum level of TAT complex was significantly lower among the Tx arm compared to the SC arm (24.8 [18.2, 38.1] vs.40.0 [25.7, 51.4]) ng/ml, p <0.001. However, the Tx arm TAT complex level was still significantly higher than either SNTCD or HbAA; p = 0.002 and 0.007 respectively (Fig 1D). Further, serum D-dimer levels were significantly lower among the Tx arm at study exit than among the SC arm (9.2 [2.8, 35.6] vs. 19.1 [5.7, 37.7]) μg/ml, p <0.001. There was no significant difference in the serum D-dimer levels between Tx arm and either SNTCD or HbAA respectively (Fig 1F).

### Change in serum levels of biomarkers from baseline, by treatment group

**Fig 2:**
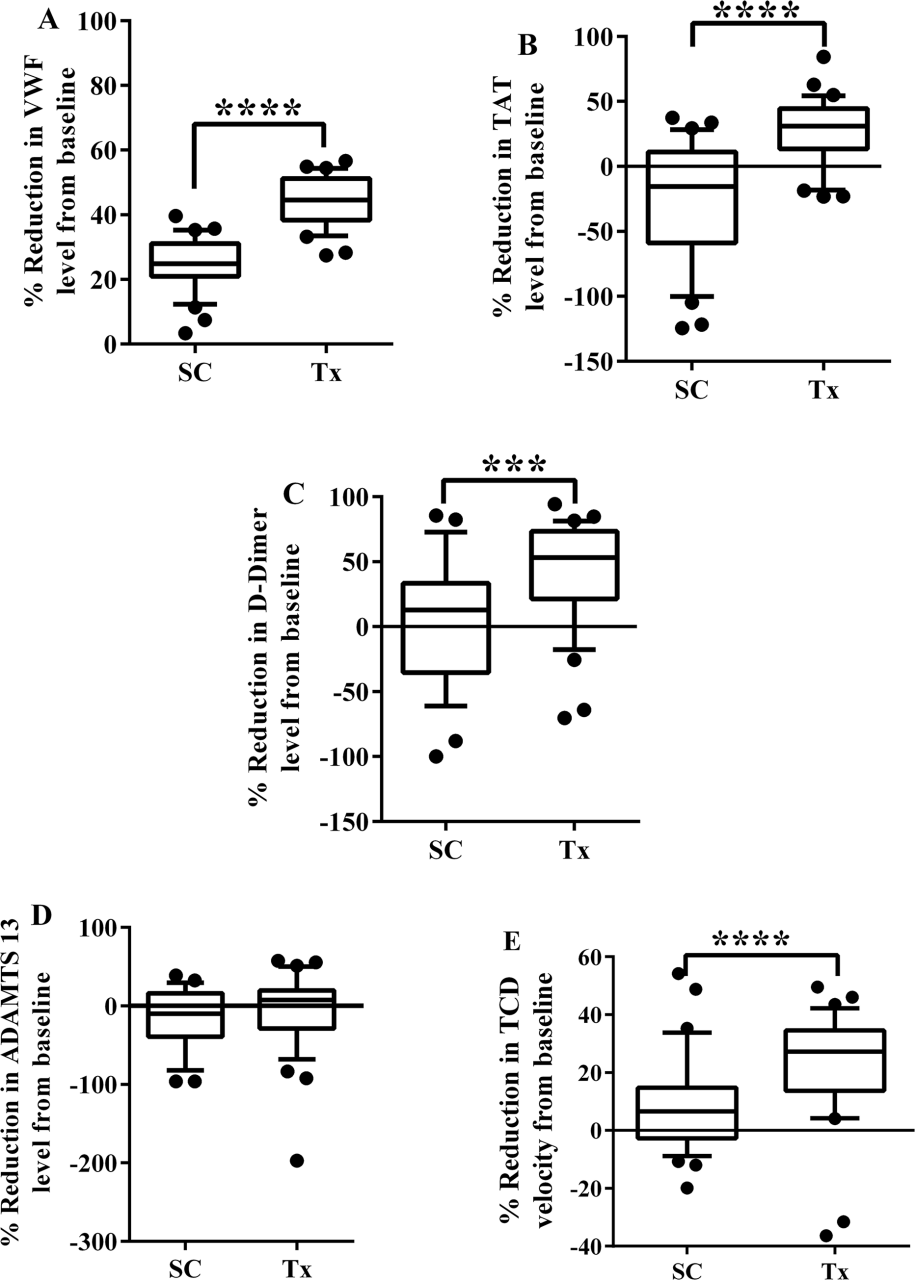
Percentage reduction in levels of coagulation biomarkers and TCD velocities in Standard Care vs. Transfusion subjects, comparing study exit to baseline values. von Willibrand Factor (VWF) (A), thrombin antithrombin (TAT) complex (B), D-dimer (C) and TCD velocity (D). SC and Tx represents sickle cell subjects randomized to the Standard Care and Transfusion arms respectively. *** = p <0.001 and **** = p <0.0001.

Since both the SC and Tx groups showed significant decrease in both TCD velocity and some of the biomarkers of coagulation activation from baseline values, we compared the percentage reduction in TCD velocity and biomarkers of coagulation activation at study exit compared to baseline in SC and TX groups (Fig 2A–2E). The percentage reduction in vWF levels from baseline was significantly greater in the Tx group compared to SC (44.5 [33.5, 54.4] vs. 24.8 [2.4, 35.2], p <0.0001). A similar pattern was observed for percentage reduction in TAT (30.9 [-18.3, 54.3] vs. -15.5 [-100.2, 28.2], p <0.0001) and D-dimer (47.1 [-223.0, 80.8] vs. 3.7 [-98.8, 71.3], p <0.001). The pattern of percentage reduction in serum levels of these biomarkers was similar to that of percentage reduction in TCD velocity in response to blood transfusion (27.2 [4.2, 42.2] vs. 6.7 [-8.9, 33.8], p <0.0001).

### Relationship of blood transfusion, TCD velocity and hematological variables to biomarker levels

**Fig 3:**
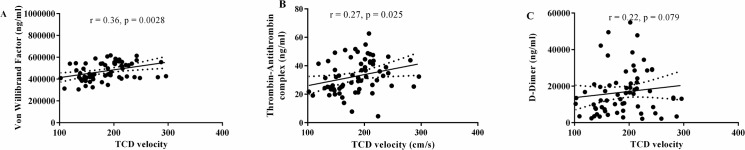
Correlation of transcranial Doppler ultrasound velocity with serum levels of biomarkers of coagulation. Serum von Willibrand Factor (vWF) (A), thrombin antithrombin (TAT) complex (B) and D-dimer (C) levels at baseline were significantly positive except for D-dimer (3C).

**Fig 4:**
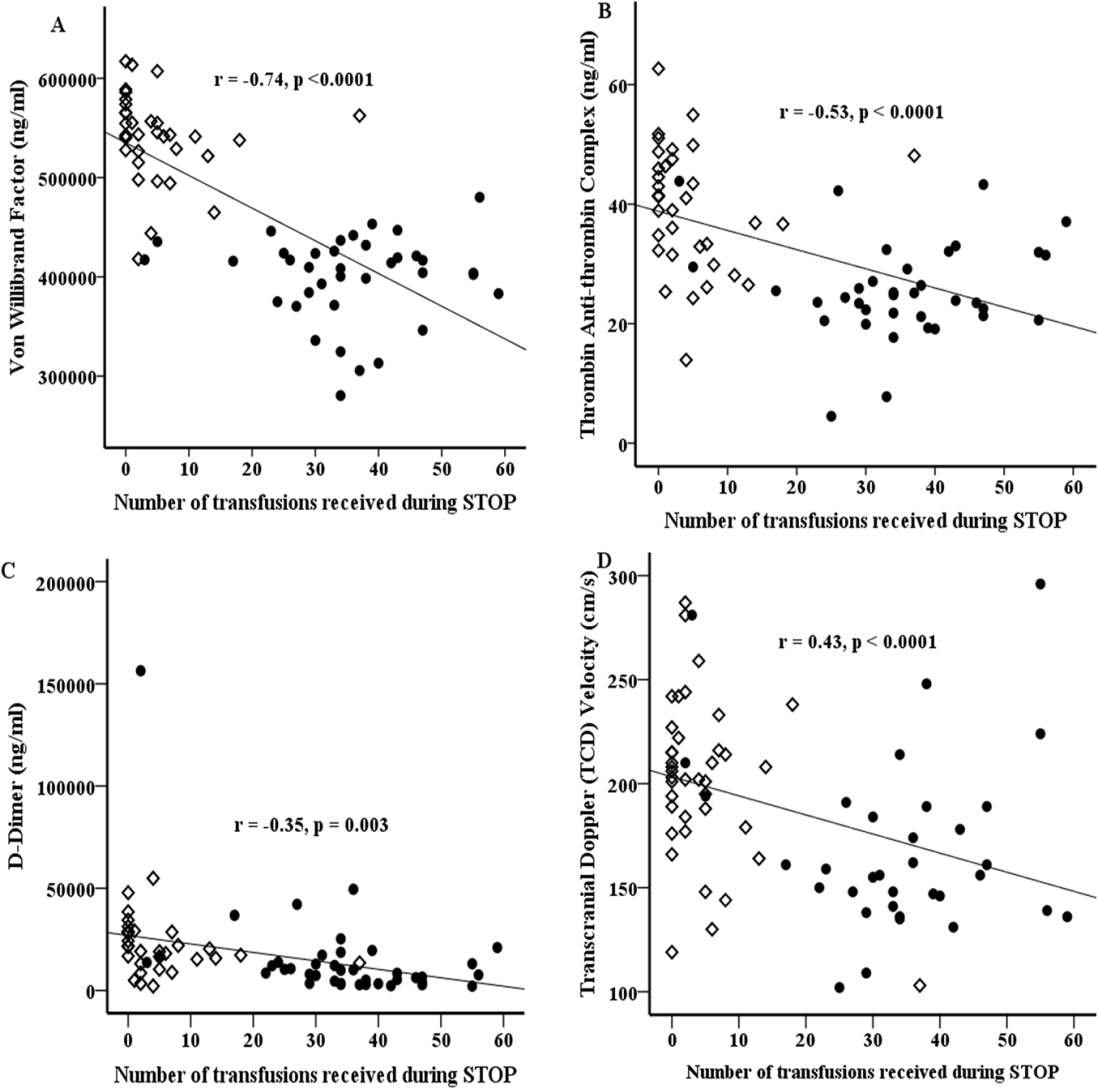
Plots of coagulation biomarker levels or TCD velocity against total number of PRBC transfusions received over the course of the trial. von Willibrand Factor (vWF) (A), thrombin antithrombin (TAT) complex (B), D-dimer (C) levels and TCD velocity (D) All 3 biomarkers and TCD velocity show significant negative correlation with number of blood PRBC transfusion. Note in the figure the almost clear separation of subjects randomized to no transfusion (open diamond) and transfusion (solid circles) arms based on the number of PRBC transfusions received. Standard care arm = Open diamond. Transfusion arm = Solid circle.

We used partial correlation to test the associations between the number of blood transfusions received, TCD velocity and hematological variables (white cell, red cell and platelet counts, also Hb and HbF level) to levels of biomarkers of coagulation activation and thrombin generation at study exit, adjusted for baseline levels. Figs 3 and 4 shows the result of those tests that were statistically significant. Fig 3A and 3B shows that there was a significant positive correlation between TCD velocity and both serum vWF and TAT levels (r = 0.36, p = 0.0028) and (r = 0.27, p = 0.025), respectively. There was a trend to a positive correlation (r = 0.22, p = 0.079) between serum D-dimer levels and TCD velocity (Fig 3C). These results show a relationship between biomarkers of coagulation activation and TCD velocity.

At baseline, vWF level was significantly correlated with hemoglobin F (r = -0.32, p = 0.006) and hemoglobin S (r = 0.38, p = 0.001) levels. Further, there was a significant negative correlation between TAT levels and WBC count (r = -0.27, p = 0.020).

At study exit, there was significant correlation between vWF and hemoglobin S (HbS) (r = 0.27, p = 0.026), HbF (r = 0.35, p = 0.003), Hb level (r = -0.40, p = 0.001) or reticulocyte count (r = 0.25, p = 0.037). Similarly, serum TAT complex showed significant correlation with HbS (r = 0.26, p = 0.028), HbF (r = 0.38, p = 0.001), Hb (r = -0.35, p = 0.003) or reticulocyte count (r = 0.38, p = 0.001). We also observed that serum D-dimer levels were significantly correlated with HbS (r = 0.44, p <0.0001), HbF (r = -0.37, p = 0.002), Hb (r = -0.45, p <0.0001) or reticulocyte count (r = 0.40, p = 0.001). There was no significant correlation between the other biomarkers tested and HbS, HbF, Hb levels or with any other hematological marker; except for that between serum sP-selectin and HbS (r = 0.25, p = 0.036) or platelet count (r = 0.024, p = 0.048). These result shows that clinical parameters related to SCD severity and hemolysis were correlated to serum levels of biomarkers of coagulation activation and thrombin generation.

When we tested whether serum levels of biomarkers of coagulation activation correlated with the number of blood transfusions received from study entry to 1 year post-trial, we noted a strong negative correlation with serum vWF (r = -0.39, p = 0.0028), TAT (r = -0.53, p < 0.0001) and D-dimer (r = -0.55, p<0.0001) levels. The pattern was similar to the correlation we observed between TCD velocity and number of blood transfusions received over the same period (Fig 4A–4D respectively).

## Discussion

In this study, we report the effects of packed red blood cell (PRBC) transfusion on biomarkers of coagulation activation and thrombin generation in children with SCD who had a high TCD velocity and increased stroke risk. We had previously reported that elevated levels of PDGF-AA were associated with increased risk of incident stroke [11] and that frequent PRBC transfusion was associated with reduction in blood levels of biomarkers of inflammation and endothelial activation [12].

We found that children with SCD at high risk for stroke had significantly elevated serum levels of biomarkers of coagulation activation and thrombin generation at baseline, essentially a worse hypercoagulable state, compared to age-matched African American children with SCD and normal TCD velocity or healthy African American children without SCD (Fig 1). The serum levels of these biomarkers tracked with TCD velocity. At study exit, there were significantly lower levels of vWF, TAT and D-dimer, along with lower TCD velocity among Transfused subjects compared to the Standard Care group. Further, correlation analysis showed that TCD velocity was positively correlated with both vWF and TAT levels. Our data suggests that coagulation activation might be an important contributor to cerebrovascular complications in SCD.

In individuals without SCD, elevated vWF levels have been associated with increased risk for stroke, as both acute stroke patients and those with carotid atherosclerosis had elevated blood levels compared to healthy controls [28]. Further, elevated D-dimer levels are associated with increased risk for stroke and cardiovascular events [29]. Thus, our finding of significantly elevated vWF, D-dimer and TAT levels among children with SCD who had a higher risk for stroke was not surprising because of these well-established association in non-SCD related cases of cerebrovascular and cardiovascular diseases [28, 29].

A similar relationship has been described in sickle cell patients with acute chest syndrome or pulmonary hypertension. Patients with SCD who had pulmonary hypertension, a history of stroke, retinopathy and increased pain frequency had one or a combination of high serum prothrombin fragment F2.1, TAT or D-dimers in addition to elevated levels of inflammatory biomarkers [8, 9]. In another study, coagulation activation was associated with SCD related small vessel cerebral vasculopathy [30].

The STOP study showed that chronic PRBC transfusions reduce TCD velocity and the rate of strokes in high risk children with SCA [18]. We found that PRBC transfusions also significantly reduced biomarkers of the hypercoagulable state. The serum levels of vWF, TAT and D-dimer levels were negatively correlated with the number of PRBC transfusions, suggesting a dose-response relationship. In addition, we found that the number of PRBC transfusion was also negatively correlated with TCD velocity. Taken together, our results indicate that thrombogenesis and a hyperactive coagulation system are likely to play a role in the pathobiology of cerebrovascular disease in children with SCD.

One of the beneficial effects of PRBC transfusions in stroke prevention may be the improvement of the procoagulant internal milieu. This assertion is supported by evidence from a number of studies. First, in a recent study, it was reported that PRBC transfusion in the STOP study patients resulted in low serum free hemoglobin level [31]. Levels of serum free hemoglobin have been found to be positively associated with levels of vWF multimers (which is procoagulant in nature) because free hemoglobin binds to the vWFA2 receptors and prevents the cleavage of vWF by ADAMTS 13 [32]. Hemoglobin inhibition of ADAMTS 13, results in high circulating concentration of vWF multimers. Secondly, there is evidence from multiple studies that repeated sickling and unsickling of RBCs in SCD, results in an over exposure of membrane phospahtidyl serine (PS). The subpopulation of PS exposed RBC in health individuals was about 0.59±19% [33], but in SCD patients, the proportion was between 2–26%. This is thought to create a hypercoagulable milieu that activates thrombin more powerfully than in non-SCD individuals [33–36]. The benefit of chronic PRBC transfusion might in part be due to a reduction in the proportion of PS exposed RBCs in SCD patients receiving such therapy.

A potential limitation of our study is that the coagulation assays were not performed on platelet poor serum, but on serum. This has a potential to impact external validity if the absolute numbers were compared to those that used platelet poor serum. However, the internal validity of our group comparisons was maintained because all of our samples were handled in the same manner. Furthermore, the uniformity of the protocol described for serum by the ELISA assay kit manufacturer ensures that our findings can be reproduced if samples treated in similar manner were used. Our samples were obtained from the STOP study bio-repository which was fully established by 1999. While it could be suggested that this is an old repository and there is risk for protein degradation, studies has shown that protein markers of hypercoagulability are stable in frozen samples over a long period of storage at-80°C [37, 38]. In this study, our samples were stored at-80°C, with care taken to avoid repeated freeze- thaw cycles (maximum of two or less). Even with prolonged storage, the STOP study samples showed much serum levels of biomarkers of coagulation activation and thrombin generation than that of the more recently drawn SNTCD or HbAA samples. Finally, we noted that there was an unexpected decrease in HbS levels at study exit compared to baseline for the SC group. We hypothesize, that subjects in the SC group might have received transfusions that were not reported or that those who received a high number of reported transfusions skew the median HbS value.

In conclusion, we surmise that the observed higher levels of biomarkers of coagulation activation and thrombin generation among subjects with high TCD velocity, together with the associated decrease with transfusion as TCD velocity decreased, support a role for hypercoagulability and thrombotic phenomenon in the pathobiology of SCD-associated cerebrovascular disease. Further investigation of the role of the coagulation system is warranted to delineate its role in development of stroke and potential application as a predictive diagnostic biomarker of stroke risk in SCD patients.

## Supporting Information

S1 File Comparison of biomarker levels between standard care and transfusion arm at baseline and study exist. At study exit that, plasma sP-selectin level was significantly higher among Tx arm than either SNTCD (p = 0.03) or HbAA (p = 0.003). Similarly, plasma sP-selecting level was significantly higher among SC arm than among HbAA subjects, p = 0.02 (**Figure D**). For reasons we could not explain, at study exit, the plasma ADAMTS 13 level was higher among the SC arm than among HbAA subjects. At study entry, the median plasma vWF/ADAMTS 13 ratio was significantly higher among Tx arm than among either SNTCD (p = 0.0009) or HbAA (p = 0.02). Similarly, baseline median plasma vWF/ADAMTS 13 ratio was significantly higher among SC arm than among either SNTCD (p = 0.002) or HbAA, p = 0.02 (**Figure G**). But at study exit, there was no statistically significant difference in median plasma vWF/ADAMTS 13 ratio between groups (**Figure H**).
(TIF)

S2 File Pairwise comparison of decrease in plasma levels of biomarkers of coagulation activation and TCD velocity from baseline. It shows that SC and Tx groups both had a decrease from baseline, with TCD velocity, plasma levels of vWF and TAT showing statistically significant decreases from baseline (**Figures A-C**). The decrease in plasma D-dimer levels from baseline was not statistically significant for either the Tx group, while the SC group actually showed an increased in D-dimer levels from baseline (**Figure D**).
(TIF)

## References

[1] Centers for Disease Control and Prevention. Children with sickle cell disease had significantly higher medical costs than those without sickle cell disease. 2012;2012

[2] Hassell KL. Population estimates of sickle cell disease in the U.S. Am J Prev Med 2010; 38: S512–S521 10.1016/j.amepre.2009.12.022 20331952

[3] Smith LA, Oyeku SO, Homer C, Zuckerman B. Sickle cell disease: A question of equity and quality. Pediatrics 2006; 117: 1763–1770 16651336 10.1542/peds.2005-1611

[4] Weatherall DJ. Hemoglobinopathies worldwide: Present and future. Curr Mol Med 2008; 8: 592–299 18991645 10.2174/156652408786241375

[5] Pauling L, Itano HA. Sickle cell anemia a molecular disease. Science 1949; 110: 543–548 15395398 10.1126/science.110.2865.543

[6] Ataga KI, Orringer EP. Hypercoagulability in sickle cell disease: A curious paradox. Am J Med 2003; 115: 721–728 14693325 10.1016/j.amjmed.2003.07.011

[7] Ataga KI. Hypercoagulability and thrombotic complications in hemolytic anemias. Haematologica 2009; 94: 1481–1484 10.3324/haematol.2009.013672 19880774 PMC2770956

[8] Ataga KI, Moore CG, Hillery CA, Jones S, Whinna HC, Strayhorn D, et al Coagulation activation and inflammation in sickle cell disease-associated pulmonary hypertension. Haematologica 2008; 93: 20–26 10.3324/haematol.11763 18166781

[9] Ataga KI, Brittain JE, Desai P, May R, Jones S, Delaney J, et al Association of coagulation activation with clinical complications in sickle cell disease. PLoS ONE 2012; 7: e29786 10.1371/journal.pone.0029786 22253781 PMC3256184

[10] van Beers EJ, Schaap MCL, Berckmans RJ, Nieuwland R, Sturk A, van Doormaal FF, et al Circulating erythrocyte-derived microparticles are associated with coagulation activation in sickle cell disease. Haematologica 2009; 94: 1513–1519 10.3324/haematol.2009.008938 19815831 PMC2770961

[11] Hyacinth HI, Gee BE, Adamkiewicz TV, Adams RJ, Kutlar A, Stiles JK, et al Serum BDNF and PDGF-AA levels are associated with high TCD velocity and stroke in children with sickle cell anemia. Cytokine 2012; 60: 302–308 10.1016/j.cyto.2012.05.017 22704695 PMC3429653

[12] Hyacinth HI, Adams RJ, Voeks JH, Hibbert JM, Gee BE. Frequent red cell transfusions reduced vascular endothelial activation and thrombogenicity in children with sickle cell anemia and high stroke risk. Am J Hematol 2014; 89: 47–51 10.1002/ajh.23586 23996496 PMC4070426

[13] Adams R, McKie V, Nichols F, Carl E, Zhang D-L, McKie K, et al The use of transcranial ultrasonography to predict stroke in sickle cell disease. N Engl J Med 1992; 326: 605–610 1734251 10.1056/NEJM199202273260905

[14] Dobson SR, Holden KR, Nietert PJ, Cure JK, Laver JH, Disco D, et al Moyamoya syndrome in childhood sickle cell disease: A predictive factor for recurrent cerebrovascular events. Blood 2002; 99: 3144–3150 11964276 10.1182/blood.v99.9.3144

[15] Gebreyohanns M, Adams RJ. Sickle cell disease: Primary stroke prevention. CNS Spectrum. 2004; 9: 445–449 15162093

[16] Hulbert ML, Scothorn DJ, Panepinto JA, Scott JP, Buchanan GR, Sarnaik S, et al Exchange blood transfusion compared with simple transfusion for first overt stroke is associated with a lower risk of subsequent stroke: A retrospective cohort study of 137 children with sickle cell anemia. J Pediatr 2006; 149: 710–712 17095350 10.1016/j.jpeds.2006.06.037

[17] Deane CR, Goss D, Bartram J, Pohl KRE, Height SE, Sibtain N, et al Extracranial internal carotid arterial disease in children with sickle cell anemia. Haematologica 2010; 95: 1287–1292 10.3324/haematol.2010.022624 20220066 PMC2913076

[18] Adams RJ, McKie VC, Hsu L, Files B, Vichinsky E, Pegelow C, et al Prevention of a first stroke by transfusions in children with sickle cell anemia and abnormal results on transcranial doppler ultrasonography. N Engl J Med 1998; 339: 5–11 9647873 10.1056/NEJM199807023390102

[19] Ware RE, Helms RW, SWiTCH Investigators. Stroke With Transfusions Changing to Hydroxyurea (SWiTCH). Blood 2012; 119: 3925–32. 10.1182/blood-2011-11-392340 22318199 PMC3350359

[20] Adams RJ, Brambilla D, Optimizing Primary Stroke Prevention in Sickle Cell Anemia (STOP 2) Trial Investigators. Discontinuing prophylactic transfusions used to prevent stroke in sickle cell disease. N Engl J Med 2005; 353: 2769–2778 16382063 10.1056/NEJMoa050460

[21] Hulbert ML, McKinstry RC, Lacey JL, Moran CJ, Panepinto JA, Thompson AA, et al Silent cerebral infarcts occur despite regular blood transfusion therapy after first strokes in children with sickle cell disease. Blood 2011; 117: 772–779 10.1182/blood-2010-01-261123 20940417 PMC3035071

[22] Asare K, Gee BE, Stiles JK, Wilson NO, Driss A, Quarshie A, et al Serum interleukin-1β concentration is associated with stroke in sickle cell disease. Cytokine. 2010; 49: 39–44 10.1016/j.cyto.2009.10.002 19900820 PMC2808442

[23] Kato GJ, Hebbel RP, Steinberg MH, Gladwin MT. Vasculopathy in sickle cell disease: Biology, pathophysiology, genetics, translational medicine, and new research directions. Am J Hematol 2009; 84: 618–625 10.1002/ajh.21475 19610078 PMC3209715

[24] Merkel K, Ginsberg P, Parker J, Post M. Cerebrovascular disease in sickle cell anemia: A clinical, pathological and radiological correlation. Stroke 1978; 9: 45–52 622745 10.1161/01.str.9.1.45

[25] Prohovnik I, Hurlet-Jensen A, Adams R, De Vivo D, Pavlakis SG. Hemodynamic etiology of elevated flow velocity and stroke in sickle-cell disease. J Cereb Blood Flow Metab 2009; 29: 803–810 10.1038/jcbfm.2009.6 19209182

[26] Prohovnik I, Pavlakis SG, Piomelli S, Bello J, Mohr JP, Hilal S, et al Cerebral hyperemia, stroke, and transfusion in sickle cell disease. Neurology 1989; 39: 344 2927641 10.1212/wnl.39.3.344

[27] Jordan LC, Casella JF, DeBaun MR. Prospects for primary stroke prevention in children with sickle cell anaemia. Br J Haematol 2012; 157: 14–25 10.1111/j.1365-2141.2011.09005.x 22224940 PMC3400704

[28] Blann A, Kumar P, Krupinski J, McCollum C, Beevers DG, Lip GY. Soluble intercelluar adhesion molecule-1, e-selectin, vascular cell adhesion molecule-1 and von willebrand factor in stroke. Blood Coagul Fibrinolysis 1999; 10: 277–284 10456619 10.1097/00001721-199907000-00009

[29] Danesh J, Whincup P, Walker M, Lennon L, Thomson A, Appleby P, et al Fibrin d-dimer and coronary heart disease: Prospective study and meta-analysis. Circulation. 2001; 103: 2323–2327 11352877 10.1161/01.cir.103.19.2323

[30] Colombatti R, De Bon E, Bertomoro A, Casonato A, Pontara E, Omenetto E, et al Coagulation activation in children with sickle cell disease is associated with cerebral small vessel vasculopathy. PLoS ONE. 2013; 8: e78801 10.1371/journal.pone.0078801 24205317 PMC3808283

[31] Lezcano NE, Odo N, Kutlar A, Brambilla D, Adams RJ. Regular transfusion lowers serum free hemoglobin in children with sickle-cell disease at risk for stroke. Stroke. 2006; 37: 1424–1426 16627796 10.1161/01.STR.0000221173.97108.01

[32] Zhou Z, Han H, Cruz MA, Lopez JA, Dong JF, Guchhait P. Haemoglobin blocks von willebrand factor proteolysis by ADAMTS-13: A mechanism associated with sickle cell disease. Thromb Haemost 2009; 101: 1070–1077 19492149

[33] Whelihan MF, Zachary V, Orfeo T, Mann KG. Prothrombin activation in blood coagulation: the erythrocyte contribution to thrombin generation. Blood 2012; 120: 3837–3845. 10.1182/blood-2012-05-427856 22968460 PMC3488894

[34] Whelihan MF, Mooberry MJ, Zachary V, Bradford RL, Ataga KI, Mann KG, et al The contribution of red blood cells to thrombin generation in sickle cell disease: meizothrombin generation on sickled red blood cells. J Thromb and Haemost 2013; 11: 2187–2189. 24119168 10.1111/jth.12423 PMC3992876

[35] Zwaal RF, Schroit AJ. Pathophysiologic implications of membrane phospholipid asymmetry in blood cells. Blood 1997; 89: 1121–32. 9028933

[36] Setty BN, Rao AK, Stuart MJ. Thrombophilia in sickle cell disease: the red cell connection. Blood 2001; 98: 3228–33. 11719358 10.1182/blood.v98.12.3228

[37] Iversen LH, Thorlacius-Ussing O. Short-time stability of markers of coagulation and fibrinolysis in frozen serum. Thrombo Res 81: 253–261 10.1016/0049-3848(95)00242-1 8822140

[38] Woodhams B, Girardot O, Blanco MJ, Colesse G, Gourmelin Y. Stability of coagulation proteins in frozen serum. Blood Coagul Fibrinolysis. 2001; 12: 229–236 11460005 10.1097/00001721-200106000-00002

